# Correction: External relationships as implementation determinants in community-engaged, equity-focused COVID-19 vaccination events

**DOI:** 10.3389/frhs.2025.1650412

**Published:** 2025-07-01

**Authors:** Ramey Moore, Jennifer Callaghan-Koru, Jennifer L. Vincenzo, Susan K. Patton, Marissa J. Spear, Sheldon Riklon, Eldon Alik, Alan Padilla Ramos, Stephanie Takamaru, Pearl A. McElfish, Geoffrey M. Curran

**Affiliations:** ^1^ Office of Community Health and Research, College of Medicine, University of Arkansas for Medical Sciences Northwest, Springdale, AR, United States; ^2^ Geriatrics, College of Health Professions, University of Arkansas for Medical Sciences Northwest, Fayetteville, AR, United States; ^3^ Nursing, College of Education and Health Professions, University of Arkansas, Fayetteville, AR, United States; ^4^ Office of Community Health and Research, University of Arkansas for Medical Sciences Northwest, Springdale, AR, United States; ^5^ Department of Family Medicine, Family Medicine Residency Training Program, College of Medicine, University of Arkansas for Medical Sciences Northwest, Fayetteville, AR, United States; ^6^ Consulate General of Arkansas, Republic of the Marshall Islands, Springdale, AR, United States; ^7^ Arkansas Coalition of Marshallese, Springdale, AR, United States; ^8^ Department of Pharmacy Practice, College of Pharmacy, University of Arkansas for Medical Sciences, Little Rock, AR, United States; ^9^ Center for Mental Healthcare and Outcomes Research, Central Arkansas Veterans Healthcare System, North Little Rock, AR, United States

**Keywords:** health equity, external networks, community engagement, community-based implementation, implementation science

**Correction on**
External relationships as implementation determinants in community-engaged, equity-focused COVID-19 vaccination events By Moore R, Callaghan-Koru J, Vincenzo JL, Patton S, Spear MJ, Riklon S et al. (2024). Front Health Serv. 4:1338622. doi: 10.3389/frhs20241338622

